# Exploring synergies between human rights and public health ethics: A whole greater than the sum of its parts

**DOI:** 10.1186/1472-698X-8-2

**Published:** 2008-01-31

**Authors:** Stephanie Nixon, Lisa Forman

**Affiliations:** 1 Department of Physical Therapy, University of Toronto, 160-500 University Avenue, Toronto, Ontario, M5G 1V7, Canada; 2 Health Economics and HIV/AIDS Research Division (HEARD), University of KwaZulu-Natal, Durban, South Africa; 3 Comparative Program in Health and Society, University of Toronto, Munk Centre for International Studies, 1 Devonshire Place, Toronto, Ontario, M5S 3K7, Canada

## Abstract

**Background:**

The fields of human rights and public health ethics are each concerned with promoting health and elucidating norms for action. To date, however, little has been written about the contribution that these two justificatory frameworks can make together. This article explores how a combined approach may make a more comprehensive contribution to resolving normative health issues and to advancing a normative framework for global health action than either approach made alone. We explore this synergy by first providing overviews of public health ethics and of international human rights law relevant to health and, second, by articulating complementarities between human rights and public health ethics.

**Discussion:**

We argue that public health ethics can contribute to human rights by: (a) reinforcing the normative claims of international human rights law, (b) strengthening advocacy for human rights, and (c) bridging the divide between public health practitioners and human rights advocates in certain contemporary health domains. We then discuss how human rights can contribute to public health ethics by contributing to discourses on the determinants of health through: (a) definitions of the right to health and the notion of the indivisibility of rights, (b) emphasis on the duties of states to progressively realize the health of citizens, and (c) recognition of the protection of human rights as itself a determinant of health. We also discuss the role that human rights can play for the emergent field of public health ethics by refocusing attention on the health and illness on marginalized individuals and populations.

**Summary:**

Actors within the fields of public health, ethics and human rights can gain analytic tools by embracing the untapped potential for collaboration inherent in such a combined approach.

## Background

The fields of human rights and public health ethics each offer frameworks that may inform normative health issues. To date, however, little has been written about the contribution that these two justificatory frameworks can make together. This article explores how a combined approach may offer a more comprehensive analysis of normative issues related to health than either approach made alone.

This discussion is timely given the range of profound health-related concerns facing both public health ethics and human rights. The wide and, in some cases, growing disparities in health and wealth both within and between countries present a formidable challenge to both fields. Health challenges facing the global poor are daunting, including: infectious disease pandemics, such as HIV/AIDS, tuberculosis and malaria; rising levels of non-communicable disease; and, limited access to adequate health care facilities, goods and services including, especially a dearth of medicines and health care workers. Indeed, many population health indices have been worsening despite commitments to health improvement in international human rights instruments and consensus agendas like the Millennium Development Goals. [1,2].

In this article, we argue that the fields of human rights and public health ethics may enjoy greater conceptual synergy than currently realized. Our effort to elaborate this synergy responds to Jonathan Mann's call for greater attention to the rapidly evolving relationships among medicine, public health, ethics and human rights in response to emergent global health crises [3]. It is notable, however, that Mann's vision was to promote human rights as a corrective to bioethics' focus on individualistic issues and autonomy to the exclusion of the social and political determinants of health. Since Mann's clarion call, public health ethics has emerged as a field of study concerned with ethical issues associated with the broader determinants of health and, increasingly, with the role of national and transnational actors in the global health sphere. International human rights law is similarly concerned with collective social welfare claims, including intra- and inter-state conduct in relation to health. Thus, both human rights and public health ethics are concerned with promoting health and elucidating norms for improved collective action in this regard.

Our analytical point of departure in this paper is not framed by adherence to any particular definition of health, but rather by the intersections between definitions of the right to health and of public health ethics. Thus, we understand the right to health as creating entitlements to access adequate health care facilities, goods and services, and the underlying determinants of health, such as food, housing, access to water and adequate sanitation, safe working conditions and a healthy environment [4]. We further understand these entitlements to place duties to progressively realize access within available resources on a broad range of actors, including primarily states but also international organizations and other non-state actors. While this implies a standard of health care relative to variable development levels, we understand this standard to be bound by a minimum essential level of health care as defined in international human rights law. Furthermore, we propose a particular notion of public health ethics that is attentive to global health concerns and power relations between rich and poor countries [5]. These definitions frame and guide our exploration of the analytical and normative contribution that these two fields can make towards improved collective action to address global health challenges.

Thus, while both human rights and public health ethics offer valuable frameworks for elaborating norms for health action, we seek to demonstrate the greater utility of a combined approach. We explore this synergy by first providing overviews of public health ethics and of international human rights law relevant to health, and second, by articulating complementarities between these two areas.

## Discussion

### Overview of Public Health Ethics

Although public health practitioners have long faced ethical challenges, the academic field of public health ethics has only recently emerged. As such, its scope and comparative position are only now being established. Callahan and Jennings have contributed significantly to the evolution of the field by proposing a four-part typology of public health ethics analyses [6]. First, they describe *professional ethics *based on professional character and ethical principles regarding trust and legitimacy in the profession. An example of this approach is the Public Health Code of Ethics [7]. Second, *applied ethics *involves reasoning from general ethical theories to inform the profession. Such an approach is exemplified by Upshur [8]. and Kass [9] who offer justificatory frameworks for public health interventions. Third, *advocacy ethics *is less theoretical but arguably the most pervasive in public health with its strong orientation towards equality and social justice [10]. Finally, *critical ethics *is practically oriented toward real-life problems, but brings larger social values and historical trends to bear. Critical public health ethics understands dilemmas not only as the result of behaviours of disease organisms and individuals, but also as resulting from institutional arrangements and prevailing structures of cultural attitudes and social power.

Along with scope and definition, public health ethics is also grappling with philosophical issues underpinning the various analyses, many of which accord with the three major themes in contemporary public health discourse: utilitarianism, liberalism and communitarianism[11]. Public health has strong roots in utilitarianism because of its fundamental focus on collective health. However, one of the critiques of this approach derives from deontological and rights-based perspectives on protecting minority rights within an overarching concern for maximizing results. Liberalism also underlies some of the major philosophical trends in public health thinking. This 19^th ^century political doctrine, strongly influenced by Kantian thinking, is concerned with issues of liberty, fairness and rights. Finally, communitarian ethics concerns the idea of a good society in which individuals exemplify appropriate virtues. There is a division within communitarianism between understanding morality to be relativistic versus a universalist approach in which ideal norms are applied across societies. This divide has relevance to public health ethics where unique cultural practices impact on health. These theories also indicate underlying linkages with human rights, which also grapple with these themes in relation to health, particularly regarding utilitarian public health measures that violate individual and collective interests.

Although many concerns articulated under the rubric of public health ethics focus on the problems of the West, one arm of discourse has promoted attention to developing country issues like infant mortality and access to essential medicines. It is within this context that some have pointed to the importance of incorporating human rights norms. For instance, Benatar *et al*. have proposed a framework for global health ethics that includes moral dimensions of public health at the international levels [12]. They have identified values beyond those espoused by Principlism (i.e., autonomy, beneficence, non-maleficence and justice) that need to be promoted to address the moral challenges posed by global health considerations. These values include human rights, responsibilities and needs; equity; freedom; democracy; and solidarity. Within this approach, they call for the linking of human rights to a broader moral agenda embracing the duty to meet essential human needs and to achieve greater social justice within and between nations. It is this arm of public health ethics that holds the most promise for grappling with devastating health concerns that are linked to political and economic determinants, such as the effects of war, forced migration and oppressive immigration policies. These concerns also foreshadow synergies with human rights.

Farmer and Campos Gastineau have appealed for public health ethics to shift its attention from health-related dilemmas in developed countries to the profound inequity characterizing health and illness in the vast majority of the world's population [13]. They further suggest that global health equity concepts should draw significantly from human rights protections of social and economic rights and concern with the poor.

This paper attempts to delve into these challenges by exploring synergies between public health ethics and human rights vis-à-vis global health concerns with a focus on how these frameworks can be mutually supportive.

### Overview of Human Rights to Health

Although human rights have antecedents in natural law and moral and political philosophy, like bioethics, the primary impetus for its modern development was the Holocaust and Second World War. These events precipitated the formation of the United Nations as a bulwark against war and state-sanctioned dehumanization and extermination. In 1948, those human rights considered to be universal were articulated in the Universal Declaration of Human Rights (UDHR)[14], a document that has become an iconic international human rights instrument. Its rights and freedoms were subsequently expanded in the International Covenant on Civil and Political Rights [15] and the International Covenant on Economic, Social and Cultural Rights [16]. These treaties have since been joined by over 100 other human rights instruments [17]. Through these iterations, international human rights have come to be characterized as universal, indivisible, interrelated and interdependent [18].

International human rights law focuses on the protection of the inherent dignity and equal and inalienable rights of all people, an idea drawing strongly from the Kantian injunction to treat every human being as an end and not as a means. This focus translates into an abiding attention to the poor, vulnerable and marginalized *- *those people routinely excluded from the benefits and opportunities of the political, economic and social mainstream. In practice, protecting dignity and equal worth requires protection of a range of civil, political, economic, social and cultural rights, with health protected as an intrinsic and instrumental part of an equal and dignified existence. This idea is captured in the UDHR, which proclaims people's right to a standard of living adequate for their health and well-being that includes medical care and other basic necessities like food, clothing and housing. Thus, while the right to health is an important free-standing right, it is closely linked to many other human rights protections contained in international treaties and domestic constitutions, including rights to life, non-discrimination, privacy and freedoms of association, assembly and movement. Theoretical arguments of the indivisibility of rights are made real in the jurisprudence of domestic courts in India and Canada, which have granted access to health care through claims made under civil rights to life, and equality [19].

Health as a free-standing human right has been entrenched in several international human rights treaties and instruments, and in each of the major regional human rights systems. In the past decade, the right to health has seen an unprecedented level of interpretation, expanding both its normative scope and identifying specific state obligations (e.g., responsibilities of governments). For example, the Committee on Economic, Social and Cultural Rights issued a General Comment on the right to health which defined the content of the entitlement under this right, as well as clarifying what state duties to progressively realize these duties would require [20]. Importantly, this comment also identified a baseline of obligations below which no state can drop irrespective of their resources, and which are not subject to progressive realization. These are a state's minimum core obligations, which are roughly consistent with essential primary health care, and include ensuring adequate and accessible hospitals and clinics staffed by adequate numbers of health care workers, and health care services, including essential medicines. These elements provide analytical precision to what governments must provide, particularly by indicating what cannot be traded-off against competing priorities. The right to health is similarly being advanced through the work of a UN appointed Special Rapporteur on the Right to Health whose work focuses on clarifying the scope of the right to health and state obligations [21].

This is not to suggest that the right to health holds a universal legal force: it holds little or no enforceability in non-democratic or autocratic jurisdictions such as China and Myanmar. Similarly, the domestic legal force of ratified human rights treaties may vary considerably, and may depend on domestic implementation of treaties as national legislation. Nonetheless, it is increasingly evident that in spite of these lacunas and variations, the right to health is enjoying greater enforceability in domestic courts due to increased ratifications of international human rights treaties, increased entrenchment in domestic bills of rights and increased judicial willingness to enforce health rights [22,23]. The right to health is, therefore, not merely a 'manifesto' right nor simply a rhetorical tool for advocacy, but an increasingly well-developed and enforceable legal right.

Nonetheless, the limitations of international human rights law must be acknowledged. This body of law is largely applicable to states and deals only weakly with the human rights duties of corporations or international organizations. Furthermore, it deals primarily with a state's responsibilities to its own population. These weaknesses are not, however, without important codicils: international law does extend human rights duties to inter-state behaviour as well as towards the populations of other states, although the binding legal nature of such duties is disputed. For example, under the right to health, states have international obligations of cooperation and assistance to enable the realization of the right to health in poorer states [24]. In addition, international legal scholars argue that the principle of solidarity (understood as a principle of cooperation with the goal of mutually beneficial outcomes amongst a world community of interdependent states) is increasingly represented in international law and state practice [25,26]. At the same time, there is a growing recognition that international human rights law imposes duties on all social actors, and not just states [27]. These elements of international law suggest that many of its limitations are becoming partially rectified through emerging conceptions of its broader global reach and application to all social actors.

However, irrespective of their legal force in any particular jurisdiction, in the sixty years of their existence as international law, human rights have assumed considerable moral and normative force, becoming seen as the "dominant moral vocabulary of our time" [28] and the only political-moral idea that has received universal acceptance [29]. Indeed, normative shifts achieved in the past century around slavery, women's right to vote, colonialism and apartheid have first and foremost been a product of rights-based social movements. Emerging evidence suggests that the use of human rights strategies in combination with social action has achieved changes in domestic governance across a "strikingly different range of regions, countries, socio-economic systems, cultures, and types of political regimes" [30]. These outcomes suggest that human rights hold a normative force irrespective of their legal status that can be mobilized to effectively shift some of the political practices that perpetuate and exacerbate current global health challenges (such as access to medicines under the World Trade Organization's trade rules).

### Complementarities Between Public Health Ethics and Human Rights

The balance of this paper articulates how an approach that combines human rights and public health ethics can make a more comprehensive contribution to the normative analysis of health issues than either approach made alone. We argue that the two approaches together offer a justificatory framework for collective action regarding the needs of the global poor that strengthens the rationale for action. Thus, the combined force of the two approaches offers to resolve some of the deficiencies of either approach taken alone, and to advance a normative framework more attentive to the health needs of the poor in all countries. In the first section, we describe three of the ways that concepts embodied by public health ethics can serve as an analytic complement to human rights on issues of international health. In the second section, we describe a range of contributions that the established field of human rights can make to the evolving discipline of public health ethics. In each case, we describe real-world health debates that can be informed by the synergistic approach of these two frameworks.

#### How Public Health Ethics Can Contribute to Human Rights

##### (a) Reinforcing the Normative Claims of International Human Rights Law

While some public health measures may be legislated, broader ethical obligations within public health function as appeals to morality and justice rather than as enforceable legal rules. Conversely, while many aspects of international human rights law are legally binding, some are more controversial and less susceptible to legal enforcement: this is true, for example, of the duty of states to provide international assistance and cooperation. Viewing this as an ethical duty in addition may bolster arguments for state compliance and contribute to its broader social and political acceptance at the level of both norms and practice.

We do not, however, suggest that public health ethics currently offers a universally applicable set of norms, nor that it offers to resolve intractable human rights dilemmas such as its cross-cultural relevance to practices like female genital mutilation. We suggest instead that the emerging field of public health ethics may offer assistance to aspects of human rights whose legal and political force is less developed, and that this complementary approach may advance improved global health action. For example, international legal scholars argue that the principle of solidarity understood as a principle of cooperation aiming for mutually beneficial outcomes amongst interdependent states – is increasingly represented in international law and state practice [31], although the content and legal force of this principle is disputed [32]. Yet solidarity is also considered a critical value underlying global public health ethics. Public health ethics can supplement a global understanding of what solidarity means for state conduct, even reinforcing its development as an international legal principle. This could expedite the lawmaking process acting in a similar way as soft, or non-binding, international legal norms and, in turn, could reinforce the norm as a primary ethical principle for global action. Viewing solidarity as a legal and ethical principle may, therefore, facilitate broader acceptance of the principle in both disciplines, and ensure the development of practical applications.

At more a practical level, the synergy between ethical and rights responsibilities is exemplified in present debates over the duties of pharmaceutical corporations with respect to access to medicines in developing countries. At present, international human rights law is only weakly applicable to corporate actors. In spite of this legal lacuna, there is a growing public call for greater corporate responsibilities regarding human rights in various areas such as labour practices, consumer safety and, most recently, access to medicines in developing countries. Such efforts are exemplified in the emergence of global corporate responsibility initiatives, such as the Global Compact [33] and the International Labour Organization's Tripartite Declaration of Principles Concerning Multinational Enterprises and Social Policy [34]. These latter processes are elucidating 'soft law' principles on human rights with which companies should comply, despite having little formal legal status.

A recent example of such a process can be seen in the release by the UN Special Rapporteur on the Right to Health of draft "Human Rights Responsibilities for Pharmaceutical Companies in relation to Access to Medicines." These duties are not articulated as peremptory duties that companies "must" engage in, but as actions they "should" undertake. The guidelines offer a framework of ethical conduct for the pharmaceutical industry in a range of areas including in relation to access to medicines in developing countries. Yet while these responsibilities are couched in the language of rights, they are more appropriately classified as ethical as opposed to legal duties. The guidelines offer the pharmaceutical industry greater precision regarding their ethical conduct in a range of areas and offer social actors a yardstick by which to measure the ethical actions of the industry. The combined effect of these guidelines may be to strengthen both human rights and ethical frameworks in this area, and to contribute towards a public conception of corporate responsibility, which, in the long run, may lead to greater legal enforceability of these duties.

##### (b) Broadening the Advocacy Framework of Human Rights

Callahan and Jennings describe critical public health ethics as locating public health issues within social, political, economic and historical contexts. This type of analysis is practically oriented to contemporary problems, but takes into consideration larger social values and historical trends. Importantly, critical public health ethics understands dilemmas as arising from institutional arrangements and power structures.

While human rights are also concerned with these issues, their specific starting point is the foundational idea of inherent dignity, worth and equal rights of all humans. While practical considerations of ways to protect human dignity may be rooted in social and historical causes, the addition of a critical public health ethics perspective may substantially broaden the scope of analysis to increase attention to the impact of institutional power relations on people's ability to enjoy human rights. For example, the eight Millennium Development Goals (MDGs) unanimously adopted by all UN member states in 2001 reflect the basic thrust of states' human rights responsibilities to enable the realization of social and economic rights such as health, both through domestic and international action. Yet, while human rights obligations provide a strong justification for wealthy countries to comply, a critical public health ethics approach seeks to locate the MDGs within broader historical, political and economical contexts, and to question, for example, why seven of the eight goals are the responsibility of poor countries with only one goal articulating requirements for rich nations.

A critical public health ethics approach can also illuminate a broader range of rationales for rich country action in fulfilling Millennium Development Goal 8: "A global partnership for development," such as equity and solidarity, and utilitarian self-interest arguments based on health, security or economic returns. Equally relevant is reasoning from critics like Pogge and Benatar *et al *who argue that the past and present policies of wealthy nations have created and maintained poverty and ill health and, therefore, that wealthy nations bear a commensurate responsibility to help alleviate these problems [35].

Locating global commitments such as the MDGs within a framework that articulates both legal duties and moral claims offers stronger justifications for their fulfilment, and provides advocates with broader arguments with which to confront non-compliers.

##### (c) Ethics as a Bridge in the "Public Health vs. Human Rights" Debate

Health issues such as HIV/AIDS have ignited divisive debates over the relative value of a public health approach "versus" a human rights approach to disease control and treatment, exemplified by the argument around patient-initiated (opt-in) versus routine (opt-out) HIV testing. This topic has received impassioned attention from both public health and human rights experts [36]. For example, public health experts have charged that:

human-rights based approaches to HIV/AIDS prevention might have reduced the role of public health and social justice, which offer a more applied and practical framework for HIV/AIDS prevention and care in Africa's devastating epidemic [37].

Inherent in this debate is a critique of human rights for being overly individualistic, which, it is argued, can detract from the population-level goals of public health. Certainly aspects of this critique are accurate–rights are individually held entitlements. However while rights claims are inherently individualistic, they are not exclusively so. A right such as health holds a strongly collective element, and the individual entitlement cannot be met without an adequate collective health care system. The more salient point for the purposes of the present discussion is the extent to which individual rights can 'trump' these collective interests, and when it is appropriate to limit individual rights or collective health accordingly. Different jurisdictions may attempt to achieve this balance in very different ways. This is animated by the contrasting approaches to balancing competing individual and public interests inherent in different national rights-models: for example, the US model is of absolute rights that trump all competing public interests, while the approach advanced in international human rights law and other important constitutional jurisdictions like Canada and South Africa is one that seeks to balance competing individual rights and collective interests having regard both to human rights principles and the impact of such limitations on both individual rights and collective interests.

Since the 1980s, human rights has articulated a series of principles – the Siracusa Principles – that indicate that that rights can be limited in service of public health provided that such limitations are both necessary and proportional [38]. Ironically, the Siracusa Principles, which are human rights norms, provide weighty support for an opt-out approach to HIV testing, which has traditionally been understood as a predominantly public health position. Nonetheless, human rights advocates have come to realize that in countries where HIV/AIDS is highly-prevalent, routine testing can be seen as both necessary and proportional provided that appropriate counselling and protection against adverse outcomes is provided. Thus the Siracusa principles offer to resolve apparent dichotomies between public health's primary mandate of protecting population health and the human rights imperative to protect individual rights [39]. These two goals are not necessarily in conflict and, in most cases, are mutually reinforcing. Articulating these principles as ethical norms may strengthen their acceptance and application by some public health professionals who view human rights as imposing unacceptable obstacles to public health practice.

This is not to argue that human rights should derive its legitimacy from the development of parallel ethical norms. Where public health professionals are unaware of the scope and utility of rights, this reflects an underlying need for more effective integration of human rights education into public and international health curricula and training. There is also the need to ensure that human rights and ethics do not operate as parallel yet disengaged bodies of thought given their shared concerns. For example, the principle of proportionality is also reflected in ethical frameworks that have sought to define appropriate restrictions on rights. [40]. Ethics, therefore, offer a language which can take into account both human rights and public health concerns and illuminate paths for action that respect the inherent positions of both field, offering to bridge the fields for those who perceive dichotomies.

#### How Human Rights Can Contribute to Public Health Ethics

##### (a) The Determinants of Health and the Responsibilities of States

Public health embraces an understanding of health as determined by far more than health care. Twentieth century public health had its historic beginning with the sanitary reform movement in the United Kingdom [41]. Over time, resources such as economic standing, education and housing as well as lifestyle factors such as diet, smoking, stress and exercise have come to be understood as factors influencing health. The 1978 Declaration of Alma-Ata described health as "a social goal whose realization requires the action of many social and economic sectors in addition to the health sector" [42]. From this perspective, the necessary preconditions of health also include those social and environmental components necessary for well-being, a view reflected in the 1986 Ottawa Charter for Health Promotion, which proposes that "the fundamental conditions and resources for health are peace, shelter, education, food, income, a stable ecosystem, sustainable resources, social justice and equity" [43].

However, debate continues to flourish about the relative significance of the various factors, particularly the importance of 'upstream,' structural factors such as socio-economic status in comparison to individualistic, behavioural factors such as exercise [44-46]. This debate is far from theoretical; the answer to the question of *what determines health *has far-reaching implications for governments in terms of policies, expenditures and programming. A critical public health ethics approach would be concerned to point out these political dimensions, noting that arguments in favour of individualistic determinants of health shift responsibility and, hence, costs from the state to individuals.

The field of human rights can bring several decades of debate over the right to health to bear on this public health dialogue on the determinants of health in at least three ways: (1) through definitions of the right to health and the notion of the indivisibility of rights, (2) through emphasis on the duties of states to progressively realize the health of citizens, and (3) through the recognition of the protection of human rights as itself a determinant of health.

First, human rights can contribute to the public health debate over the relative value of upstream versus downstream (or distal versus proximal) determinants of health by offering a perspective that explicitly encompasses structural, system-level forces.

This is implicit in article 25.1 of the Universal Declaration of Human Rights discussed above, which affirms:

Everyone has the right to a standard of living adequate for the health of himself and of his family, including food, clothing, housing and medical care and necessary social services.

This conception of health as part of a fundamental developmental package is reflected in the UN Social Rights Committee's General Comment 14 interpreting the right to health, which explicitly acknowledges that the attainment of health depends not only on access to appropriate health care, but also to the underlying determinants of health. Furthermore, as discussed above, the right to health is understood as indivisible from other rights. That is, the right to health may only be fully achieved by realizing other human rights, such as "rights to food, housing, work, education, human dignity, life, non-discrimination, equality, the prohibition against torture, privacy, access to information, and the freedoms of association, assembly and movement". The notion of indivisibility can contribute to the current health policy debate around determinants of health by reinforcing the central role of broader structural factors related to power and oppression in society.

Second, human rights obligations are obligations of states toward their citizens, either directly or indirectly through the regulation of third parties. At a time when many developed country governments are reducing public expenditures on health, and after decades of structural adjustment programmes that have forced the same neoliberal reasoning on developing countries, a refocus on states' legal obligations to progressively realize the right to health of all citizens offers added ammunition in both advocating for public health and, where necessary, litigating for specific health care services. This has particular salience for the ongoing debates over what constitutes healthy public policy, including the role of the private sector in delivering health care. That is, public health ethics analyses of public policy for health in both affluent and developing countries can be informed by human rights doctrine regarding the ultimate responsibility for health resting with governments in contrast to the alternate perspectives that view health as a commodity that ought to be regulated by the market.

Finally, public health ethics is concerned with identifying and advancing ideas about what ought to be done to improve the health of societies. Mann's thesis about the interconnectedness of health and human rights contributes to the understanding of what makes people healthy or ill [47]. This recognition that the protection of human rights is itself an important determinant of health is largely absent from the discourse on determinants. However, this has been shown to be a crucial factor in health promotion and disease prevention in contemporary problems like reproductive and sexual health [48]. Furthermore, the recognition of human rights as a determinant of health opens up avenues for intervention in the pursuit of improved public health that may not have been realized in the past.

This insight can also help address the current debates over the best approaches to prevention programming in HIV/AIDS. Emphasis on the ABC (abstinence, be faithful, use condoms) approach as a preferred framework for HIV/AIDS prevention programming neglects issues of power and abuse that preclude many people (especially women) from being able to make their own decisions to use ABC. Understanding the protection of human rights as itself a determinant a health, and in this case a determinant of a woman's ability to protect herself from HIV exposure, provides an important balance to the debate.

##### (b) Addressing the Health Concerns of Marginalized Individuals and Populations

The "10–90 Gap" describes the fact that only 10 percent of the world's health research resources are spent on 90 percent of the world's health problems, and *vice versa*. While public health issues in developed countries deserve ethical scrutiny, this preoccupation has precluded meaningful engagement with health issues relevant to the vast majority of the global poor.

Early writing in public health ethics has risked repeating this unjust pattern by dealing almost exclusively with issues of relevance to the rich West. However, the scope has begun to widen and one may be optimistic about the field's potential to robustly tackle issues of global health as it continues to grow [49]. Human rights may contribute to redressing this imbalance by bringing its fundamental concern with people who are marginalized and vulnerable to bear on the evolution of public health ethics, encouraging the latter to meaningfully address global health problems, and acting as an important corrective to the chronic neglect of issues facing the world's most vulnerable populations.

## Summary

This paper has illustrated a range of ways in which the fields of public health ethics and human rights offer complementary approaches to health concerns (see Table 1). Our analysis is less concerned with identifying distinctions between the two fields than to identify where overlaps between the two fields offer practical analytical tools to each other. As a relatively mature field, human rights offers the nascent field of public health ethics the benefits of increasingly well-developed notions of state responsibility with respect to health, and an obligatory legal framework for action. Public health ethics, on the other hand, offers human rights a strengthened ethical framework for action, broader justifications for claiming cooperative action in relation to health, and increased acceptance of collective ethical duties towards global public health.

**Table 1:** Complementarities between public health ethics and human rights

*How public health ethics can contribute to human rights:*	*How human rights can contribute to public health ethics:*
By reinforcing the normative claims of international human rights law By strengthening advocacy for human rights By bridging the divide between public health practitioners and human rights advocates in certain contemporary health domains.	By contributing to discourses on the determinants of health through: a. definitions of the right to health and the notion of the indivisibility of rights b. emphasis on the duties of states to progressively realize the health of citizens c. recognition of the protection of human rights as itself a determinant of health By refocusing attention on the health and illness on marginalized individuals and populations.

Actors within the fields of public health, ethics and human rights can gain analytic tools from the respective contributions of both paradigms and the untapped potential for collaboration. Without strict and enforceable legal regulation of global conduct affecting health, the contribution of rights and ethical standards to achieving more equitable outcomes should not be underestimated. Nor should the contribution of "soft" ethical norms be underestimated for their capacity to contribute to the creation of "hard" binding legal rules on global health. In this light, public health ethics and human rights should be seen as complementary projects, and should become core precepts of the public health enterprise.

## Abbreviations

Millennium Development Goals (MDGs)

United Nations (UN)

Universal Declaration of Human Rights (UDHR)

World Health Organization (WHO)

## Competing interests

The author(s) declare that they have no competing interests.

## Authors' contributions

Each of the authors contributed equally to developing the ideas in this argument and to writing the article.

## Pre-publication history

The pre-publication history for this paper can be accessed here:


